# Walking the tightrope: how rebels “do” quality of care in healthcare organizations

**DOI:** 10.1108/JHOM-10-2018-0305

**Published:** 2019-11-07

**Authors:** Iris Wallenburg, Anne Marie Weggelaar, Roland Bal

**Affiliations:** Erasmus School of Health Policy and Management, Erasmus Universiteit Rotterdam, Rotterdam, The Netherlands

**Keywords:** Organizational performance, Accounting, Quality healthcare, Rebels

## Abstract

**Purpose:**

The purpose of this paper is to empirically explore and conceptualize how healthcare professionals and managers give shape to the increasing call for compassionate care as an alternative for system-based quality management systems. The research demonstrates how quality rebels craft deviant practices of good care and how they account for them.

**Design/methodology/approach:**

Ethnographic research was conducted in three Dutch hospitals, studying clinical groups that were identified as deviant: a nursing ward for infectious diseases, a mother–child department and a dialysis department. The research includes over 120 h of observation, 41 semi-structured interviews and 2 focus groups.

**Findings:**

The research shows that rebels’ quality practices are an emerging set of collaborative activities to improving healthcare and meeting (individual) patient needs. They conduct “contexting work” to achieve their quality aims by expanding their normative work to outside domains. As rebels deviate from hospital policies, they are sometimes forced to act “under the radar” causing the risk of groupthink and may undermine the aim of public accounting.

**Practical implications:**

The research shows that in order to come to more compassionate forms of care, organizations should allow for more heterogeneity accompanied with ongoing dialogue(s) on what good care yields as this may differ between specific fields or locations.

**Originality/value:**

This is the first study introducing quality rebels as a concept to understanding social deviance in the everyday practices of doing compassionate and good care.

## Introduction

Quality improvement in healthcare is increasingly concerned with the standardization of professional behaviour and organizational routines. Starting with the “discovery” of practice variation in the 1970s, methods such as clinical guidelines, accreditation, quality registries and performance indicators have been developed to make healthcare practices increasingly measurable, comparable and governable (Bonde *et al.*, 2018; Wallenburg, Quartz and Bal, 2019; Weggelaar-Jansen *et al.*, 2018). This “system” approach to healthcare has been fostered by the release of the “Institute of Medicine (2000)” report in and its numerous follow-up accounts about the importance of improving quality of care through building organization-based systems of quality control and improvement. A striking example is the method of “root cause analysis”, a systemized procedure for investigating clinical errors that have quickly spread as an optimal and universal practice of preventing (future) medical errors as it detects organizational risks and encourages mutual learning (Nicolini *et al.*, 2016). It is generally believed that “best” practices, such as root cause analysis, and subsequent implementation of those best practices in other organizations should guide the way to better and safer care (Zuiderent-Jerak and Berg, 2010). This system approach (also termed “Safety I”) is, however, criticized for its strong focus on organizational solutions to clinical risks, overlooking the dynamics and uncertainties that characterize clinical work (e.g. Liberati *et al.*, 2019; Dixon-Woods *et al.*, 2009; Wallenburg *et al.*, 2013). Furthermore, it is argued that focusing on risks and errors neglects (and fails to appreciate) the creative and tacit work professional carry out in daily clinical practice that prevents instead of cause clinical errors (Mesman, 2012; Waring and Bishop, 2010). These scholars argue for a Safety II approach focusing on “what goes right” and further improve quality of care by learning from those valuable examples (Hollnagel *et al.*, 2013).

Likewise, recent emergence of scandals in healthcare has induced a public outcry to focus on optimal care. The English Mid Staffs scandal is a case in point. In the Stafford hospital, the later Francis Inquiry documents, patients severely suffered from a lack of care, compassion and humanity. The scandal, which was widely covered in the media, makes a plea to (re)focus on patients’ well-being and particularly on advancing compassionate care (Singleton and Mee, 2017). It draws attention to individual professionals and clinical groups that have valuable and “deviant” ideas about improving care. This movement – expressing itself through various initiatives like “Q” (Pope, 2017) and “breaking the rules” in the UK and the USA (Berwick *et al.*, 2017) with similar initiatives in the Netherlands (Kiers, 2018) – criticizes the focus on performance systems and “rules” over private initiatives to deliver high-quality care (see also Liberati *et al.*, 2019). It underscores personal and cultural deviance that is deemed crucial to come to new ways of providing patient-oriented care, and seeks to learn from those initiatives and practices to improve quality of care more widely.

In this paper, we aim to contribute to this literature by ellucidating how “good” care is constitued in deviant practices of hospital care in the Netherlands, scrutinizing what is considered good care and recognizing that what “good” is may differentiate between specific situations and among professionals and managers. We are interested in how healthcare practitioners (i.e. medical doctors and nurses) account for their (deviant) work in the light of a persistent system-oriented focus on quality control. We build on ethnographic research in three deviant hospitals practices (which we will term “clinical microsystems”) in the Netherlands. The central question guiding this paper is: how do healthcare professionals craft alternative forms of care delivery and how and to whom do they account for their practices?

The paper proceeds as follows. In the following section, we outline the literature on social deviance in healthcare. We then explain our research approach, providing also a background account for the Dutch case. We then present our findings, discussing three themes that emerged from our findings: building clinical microsystems; contexting; and dealing with organizational rules. In doing so, we present the “quality rebel” as an example of deviant practitioners that aim to provide optimal care in original and locally resonant ways. We end with a discussion and conclusion in which we relate our findings on rebellious care to contemporary policies of improving quality of care, suggesting policy and management implications.

## Social deviance in healthcare

In literatures on quality of care, “positive deviance” has been portrayed as a possible way out of the frequent remedy of further regulation and standardization. Opposed to a problem-focussed approach – meaning identifying and eliminating threats to a high level of performance – positive deviance presumes that knowledge about “what works” are available in existing organizations that demonstrate consistently exceptional performance (Bradley *et al.*, 2009; Morrison, 2006). The positive deviance approach is based on the premise that every community has individuals or groups whose uncommon behaviours and strategies enable them to find better solutions to problems than their peers or external experts (Singhal and Bjurström, 2015). Positive deviants are pointed out as those social actors possessing the reflexivity, innovativeness, agency and spaces to act otherwise and to find different institutional solutions to do better (Singhal and Bjurström, 2015). They dare to “rock the boat and stay in it” (Bevan, 2013). Positive deviants (or “quality radicals” as Bevan calls them[1]) tend to shake up the organization, but do not intend to harm or leave the organization, rather seeking to care for the organization and its purposes (i.e. caring for patients). Such “betterness”, it is contended, is often situated in the mundane and simple micro-actions carried out in everyday practices. An example is a nurse who uses her knuckle (not her fingertips) to press the elevator button in order to avoid contamination, seeking to reduce the spread of (possible) dangerous infection (Singhal and Bjurström, 2015). The positive deviance literature demonstrates the intelligibility of small and meaningful practice variation that engenders larger effects.

According to this positive deviance view, positive examples or best practices should be “unearthed”; they must be carved out of day-to-day practices and spread among others, promoting wider uptake of discovered practices within an industry and, ultimately, provoke large-scale change (Bradley *et al.*, 2009). While this approach, on the one hand acknowledges the “bottom-up” development of quality of care, on the other it denies the situatedness of such approaches, seemingly falling in the same trap as the “top-down” approaches it criticizes. That is, by focussing on the substance of the creative ways of working that positive deviants develop and then “rolling them out”, this literature risks rendering “positive deviance” the next object of standardization and performance management (cf. Waring, 2013).

In this paper, we aim to underscore a situated approach to social deviance and quality of care, highlighting how social actors are continuously confronted with quality issues that require situated responses to improving or maintaining high-quality care. Rather than seeking solid system solutions that can be standardized and implemented elsewhere, we wish to shed light on quality care as emerging and collective knowledge practices, and as a way of doing (cf. Clegg *et al.*, 2005; Mesman, 2012; Liberati *et al.*, 2019). As such, we aim to contribute to a situated understanding of quality thinking in organizations and organizational theory. We seek to shift attention from the universal and controllable to the fragmented and creative, asking how such an approach could fit into the contemporary felt need to control risks and prevent organizational reputation damage (Tarrant *et al.*, 2017; Power *et al.*, 2009). This also means that, rather than simplifying the actions of rebel groups to the elements that could be implemented elsewhere, we want to stay with the complexity of their practices, looking for the ways in which they “do” quality care given these complexities (cf. Tsoukas, 2017).

To that end – and based on our ethnographic research on doing what would be seen as “good” care in hospital practice in the Netherlands – we coin the concept of “(quality) rebels”. Rebels, we learn from movies, songs and novels, are those who act against authorities and break with established rules that hamper their desired way of living. We intend to elaborate on this idea of rebels, given that rebels seek to make sense of rules and established systems, while also seeking to work around them if they feel they can (better) achieve their goals in distinct ways. Furthermore, and learning from cases of severe medical failure in which clinicians ignored regulations to promote safe care, or even purposefully harmed patients (the so-called “bad apples”, cf. Dixon-Woods *et al.*, 2011), we are interested in how and to whom quality rebels account for their (deviant) quality work. Hence, we do not aim to revisit old-school professionalism characterized by authority and autonomy, but rather seek to articulate a situated and dynamic approach of doing and accounting for deviant forms of doing optimal care.

## Methods

### Healthcare quality management in Dutch hospitals

Like in many other countries, healthcare quality in the Netherlands has become increasingly system-based since the early 2000s onwards. Focus has shifted from professional-oriented quality regulation, primarily based on public trust, to formal, usually hospital-based regulation with state-based regulatory agencies. Among other reforms, the Dutch healthcare inspectorate has introduced a set of performance indicators to monitor and steer on healthcare quality. Typically for the corporatist nature of the Dutch healthcare system (e.g. Helderman *et al.*, 2005), this set of indicators is developed in close collaboration with the associations of physicians, hospitals and nurses’ association (Berg *et al.*, 2005; Wallenburg, Mol, Harmsen and De Bruyne, 2019). The set of performance indicators is central to quality regulation in hospitals (also because it is used by the media to compose yearly hospital rankings), yet it is now accompanied by many more sets of performance indicators developed by, among others, patient associations and healthcare insurers. In addition to the performance indicators, hospitals are subjected to accreditation systems and national quality regulations, for instance, on the safe distribution of medicine, safe practices in the operating theatre and the like. Furthermore, professional associations and the National Health Care Institute (a state agency that regulates the basic benefit package for health insurance and sets quality standards) play an important role in the development of clinical guidelines, which hospitals are expected to incorporate into their quality policies. In the past decade, all hospitals have heavily invested in hiring well-trained quality staff and building hospital quality systems – for instance, by adopting the method of root cause analysis as explained earlier. The rise of healthcare quality management has been celebrated, as it has been shown to reduce the number of medical errors and related deaths. On the other hand, it has also been criticized for having induced a lot of “red tape” (Bozeman and Anderson, 2016), for being mere impression management rather than an advance in patient safety, and for demotivating healthcare professionals who are overloaded and feel that time is moving away from their “real job” – that is, caring for patients (Kiers, 2018).

### Research approach

**Figure 1:**
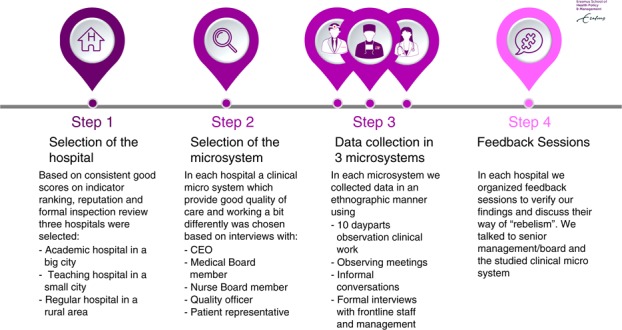
Research design and data collection

Learning about rebels and their practices demands in-depth empirical research. This paper builds on an ethnographic study in three hospitals in the Netherlands (November 2016–November 2017). We used a practice approach of observing rebels in their everyday work practices, interrogating ways of acting, strategies and experiences. The research was carried out in four phases: hospital selection; identification and selection of positive deviant groups; observing and interviewing participants; general focus groups and interviews with hospital executives, quality managers, middle managers and clinicians. The data collection strategy is displayed in Figure 1.

Hospitals were selected purposefully. We selected hospitals in different geographical regions that had consistent high scores on the yearly hospital rankings, had solid reputations among healthcare managers and researchers, and that we were already familiar with from various former research projects. In other words, we sought hospitals that were known as high-quality among those well-experienced in the field. We approached six hospitals. Three declined because they stated that they were already too busy (two of them were currently subject to formal accreditation processes). In the three participating hospitals, we first interviewed a hospital executive and executive director, a representative from the nurse staff and the medical staff to get an impression of deviant “rebel” groups in their hospitals (see Table I for an overview of interviewees in the different phases of the study). This would later help us conceptualize the rebels and rebel groups. So, rather than having a clear-cut view of what or who we were looking for, we searched for high reputational groups. As a part of this conceptualizing work, we questioned the interviewees about “places” (wards, clinical groups and networks) in their hospital with high-quality care and where care was done “a bit differently” and not always in alignment with hospital policies. We also inquired why those specific places (in all cases, clinical wards) were pointed out to us, seeking to get a better understanding why certain actors are seen as rebels, with what their deviance is compared to (i.e. which written and unwritten rules, approaches and culture they were perceived to breach) and why they were still pointed out as “good” or “trustworthy”. On this basis, three rebel groups across the three hospital were selected: a nursing ward for infectious diseases, a mother–child care department and a dialysis unit. In the analysis, we will refer to them as Hospital A, B and C, respectively.

In all wards, we spent 10 part days (over 120 h in total, 40 h per hospital) observing clinical processes as well as meetings, shifting our presence between morning, day and night rounds and including one weekend. In each unit we also held formal interviews with five members of staff (managers, nurses and medical specialists). All interviews were fully transcribed and observations were written down immediately after (and sometimes during) observation hours. After our initial analysis, we held member-check events in all hospitals – interviews and, on request of two hospitals (Hospitals B and C), two focus groups, including the leaders of the rebel groups as well as most of the people we had interviewed in the first phase. In the focus groups, team managers and representatives from various professional groups were also present because they sought to learn from the rebel groups we studied. Focus groups were organized with the explicit permission of the rebel group we had studied in the particular hospital. Hospital ethical approval was formally requested, but the project was deemed exempt.

### Analysis

Analysis was undertaken using the method of abductive analysis (Tavory and Timmermans, 2014). Typically, abductive analysis combines deductive “theory driven” and inductive “data driven” coding, letting those codes and outcomes “speak to each other”. This iterative way of analyzing data allows for an explorative approach of finding relevant patterns in the data (Stoopendaal and Bal, 2013). Following the abductive analysis method, we coded our empirical data (interview transcripts and observation reports) inductively, constantly comparing our grounded codes with the theoretical concepts derived from the deviance literature and compassionate care debate outlined above. Using the literature, we first developed codes that we discussed in our research groups (comprising all authors). We then read through and discussed our data, defining additional codes that were once again discussed. We then coded our data, sharing results and discussing them in cases of disagreement. Coding data were primarily undertaken by the first two authors; and discussed with the third author in cases of disagreement, and to deepen the analysis. The following section includes exemplary data excerpts which were translated from Dutch to English. All quotes have been anonymized.

## Quality rebels at work

### Clinical microsystems of doing quality work

The abductive analysis revealed how rebels build and rebuild clinical microsystems through peer selection and materializing their normativities about what “good” care yields, flexibly relating to their organizational and policy contexts. We first describe how rebels constitute quality of care and the normativities this involves. We then describe how rebels create contexts for doing quality work, and the boundary work they engage in. We show how rebels obey, bend and ignore hospital quality procedures and how they create spaces for doing so, also envisioning the dangers this may produce. In all cases, quality of care and in particular quality for individual patients was a centrally defining motivation and element. We observed that considerable effort was put in constituting the right care for a particular patient, as is illustrated in this excerpt:

During the morning report a house officer discusses a neonate suffering from a drip line sepsis. The baby has had three days of antibiotics in a row, and following the protocol blood measurements must be done today. The house officer announces the clinical procedure reading from his list. The fellow resident, who is chairing the meeting, interrupts: “We’re not going to tease him (the baby) any longer, we won’t do the measurement”. In the discussion that follows she argues that the baby is too small (only 600 grams) and she wants to save blood. She furthermore states that the baby has had enough stress, being too high in his discomfort scores already. “He needs a day off”.

(field notes Hospital B)

This excerpt elucidates the clinical tinkering (Mol *et al.*, 2010) that was central to all cases in the study. Situated patient needs, or assumed needs, social circumstances and quality of life vs clinical protocols and guidelines were constantly weighted and discussed to figure out the “right” care for this patient. Such tinkering is embedded in the clinical microsystems rebels build. The concept of clinical microsystems was coined by Nelson *et al.* (2002), pointing out small, functional, front-line units of care provision in which patient and providers meet and make up the care provided. In this research, we use the clinical microsystem concept to point out the clinical environment; that is, the practitioners, instruments, architectural context, patients and their relatives and the caring craft work (Davies and Horst, 2015) this involves – as well as the normativities embedded in these, and evident through their breaches.

The rebel clinical microsystems we observed are constructed through establishing working routines and a shared understanding of what “good” care looked like. Working routines are highly institutionalized and create a stable infrastructure of care provision. In Hospital A, for example, frequent hand-overs were carried out in which the nurse manager played a central role discussing the clinical situation of all admitted patients with the nurses and physicians, checking whether certain procedures had been conducted, and signalling possible changes in a patient’s clinical condition. Patients admitted to this ward, the nurse manager contended, were often vulnerable and clinically unstable due to their disease or drug addiction, requiring close observation and in-depth clinical knowledge. The nurse manager in Hospital A used his clinical experience to train and supervise the nurses as well as the medical residents following a routine of interrogating them about a patient’s disease(s) and social circumstances and stimulating discussions about these issues. This also emerged from the way quality initiatives were carried out. Quality improvement projects were often the result of encountered needs or difficulties in daily practice, which is illustrated by the following example, derived from Hospital C:

One of the dialysis nurses receives an email from the manufacturer, announcing that the labels of acid containers are about to change. Whilst the solutions used to have their own unique names, this will be changed to generic names. The particular solution will be labelled AC-F211.5, which is very similar to another solution being used, namely AC-F211,15. The nurse discusses the announcement during the coffee break, expressing his worries about likely future mistakes. The other nurses agree. They immediately start to brainstorm about possible solutions. A nurse suggests to color the containers. After the break, the nurse who got the email phones the manufacturer to explain the problem and possible solutions. Subsequently he initiates a set of activities to prepare the revisions coming up, amongst others by informing the chair of the quality committee to revise current protocols and adjust the dialysis machines.

(field notes Hospital C)

This excerpt conveys the pro-active way of handling quality of care that typifies the rebels we studied. Proactivity had precisely been the reason these particular clinical groups were pointed out to us as strong examples of doing healthcare quality in first instance; they are groups that dare to experiment to meet the needs of their patients[2]. These examples, among many from which we could have chosen, illustrate that rebels “do things differently” and follow their own convictions of what constitutes “good” care, also incorporating external stakeholders and their practices – something we will point out as “contexting” in the following section. Hospital C again offers a prominent example, where managers and practitioners were busy transferring dialysis care to patients’ homes:

The practitioners and managers contend that dialysis should take place at home, where patients live their lives with their disease. Hospitalization, a common phenomenon in the case of chronic patients and particularly those who need frequent treatment, should be opposed in order to improve patients’ well-being. To this end, clinical managers set up arrangements with the health insurer and telecom company to allow care at home, and invested in electric cars and bicycles to enable the nurses to visit (and care for) their patients at home. Patients are requested to provide some kind of a desk or table for the nurse to work as dialyses treatment takes a while. During this time the nurses work on their quality improvement projects.

(field notes Hospital C)

This excerpt demonstrates how practitioners’ opinion of what constitutes optimal care in a particular situation (i.e. home dialysis to steer independency and self-support) is materialized in purchasing cars, bikes and tables to enable dialysis at patients’ homes. Furthermore, it reveals rebels’ normativity and normative work of what they feel “good” care is and how this should be organized. Caring involves the patient and their relatives, the employees and the physical environment in which care and work is conducted. In all three settings, the design of the unit (paintings, coloured walls that differed from other departments) and facilities related to Information and Communication Technologies (ICT) were optimized and taken care of by the staff themselves. Sometimes this involved side-stepping hospital policies, like the orange painted wall of the nursing post in Hospital A, to facilitate – in this case “brighten up” – the environment. The environment in all three cases was something rebel groups were proud of and was actively cared for – underscoring their creative and deviant approach of doing “good” care.

Rebels, this first section demonstrates, build clinical microsystems that are social, material and normative. This building of microsystems involves constituting boundaries and taking care of them: rebels set boundaries to define their clinical field in which care is designed and carried out, both within the hospital and outside – for example, in the above-mentioned cases of the acid container and the homes of dialysis patients. They seek to design their microsystems according to their convictions of what optimal care yields, and are prepared to put considerable effort into making them work. In doing so, quality rebels put the quality of life of their patients on central stage, organizing care around how they feel quality of life can be best improved – and, if necessary, working around organizational rules in order to accomplish this. In the following section, we examine how rebels cross the boundaries of their clinical microsystem to relate to the specific organizational and policy contexts in which they conduct and construct their work.

### Creating contexts for doing quality care

Clinical microsystems are connected to various “outside” stakeholders both inside and outside the hospital. Responses to external stakeholders, in particular, indicate the entrepreneurial tenets of quality rebels:

Baxter, an international company that manufactures machines and drugs for dialysis patients, has developed a new dialysis machine. The dialysis ward of Hospitals C is one of the first departments in the world that is going to test the new machine. The manager of the department and the chair of the working group guiding the introduction of the new technique (a ward nurse), meet up with a company’s agent. Together they explore encountered difficulties and figure out possible solutions. The manager suggests development of a protocol which can also be used by other hospitals. Baxter, in turn, promises to construct a logistic validation model to enable a smooth and fast transition for patients, to prevent admission to the ward. This is all discussed in a manner that shows a relationship – not as supplier and buyer – but as partners who are collaborating over their own organizations to provide the best care possible for patients.

(Field notes Hospital C)

The above excerpt demonstrates how rebels collaborate with external stakeholders to design care processes, indicating proactivity and creativity as well as willingness to be visible and to make a tangible difference to the quality of a patient’s care. Such stakeholders are referred to as partners, signifying equality and the importance of knowledge sharing and collaboration. In Hospitals A and B, managers also built external networks to improve care. In Hospital B, one of the medical doctors held a position of Chair at a faculty for industrial design of the neighbouring technical university – something quite exceptional for a non-university hospital. The close connection with the university enabled practitioners to participate in research projects and to play an entrepreneurial role in searching for innovative technical solutions to clinical problems:

The physician explains that his team is developing an electronic pillow that simulates the heartbeat, smell and lung movements of the mother, which gives a baby in an incubator a feeling of intimacy, comfort and safety. Such feeling stimulates the production of oxytocin, which in turn stimulates brain development.

(Field notes Hospital B)

In Hospital B, a substantial number of medical residents, nurse practitioners and medical staff had also been participating in scientific research. The physician underscored the importance of the connection with the university because this was perceived to create an atmosphere of care improvement, encouraging a culture of innovation and ambition. Rebel leaders play a crucial role in creating connections with relevant stakeholders and developing opportunities for clinical development, something which can be described as contexting (Asdal and Moser, 2012) – the mobilization of contexts to pursue one’s goals. Contexting includes the creation of conditions, such as establishing trust or gaining financial resources, to get the work done, as well as safeguarding legitimacy to achieve aimed-for goals.

We found a striking example of contexting in Hospital C, where the health professionals set in motion various initiatives to improve dialysis care, particularly by stressing the importance of self-care, as was indicated above. The shift to the home as a place of dialysis care prompted a more central role for general practitioners (GPs), who had to be trained in the treatment of patients with kidney disease:

We need to do this together with the GPs, otherwise we won’t succeed. I don’t have the patience, but doctor (X) does, with his tactile appearance. If he does the job it will work out much better.

(interview medical specialist, Hospital C)

Hence, contexting is about networking and encouraging others to act in line with rebels’ practices of caring. This requires sensitive work in getting to know the other, and knowing how to influence outside practices, like the GPs described in the excerpt above. Contexting also involves stakeholders in the hospital itself. Members of the current clinical microsystems participated in hospital-wide working groups and quality committees, often upon their own initiative. Sometimes participating was done as a way of “scut work” (administrative work) (Huising, 2014), creating legitimacy and trust as well as a way of staying in touch with the wider hospital organization. At other times, participating in cross-departmental activities was considered an opportunity to actively influence hospitals policies. This was evident, for example, when guidelines or ICT tools had to be developed that touched upon the microsystem’s daily routines, such as adjustments in the structure of the electronic patient file or the design of a performance management tool.

In short, rebels actively influence their working environment by ways of contexting; they create and nurture connections with outside stakeholders to innovate and establish new working practices. Contexting requires active, outreaching and sensitive work. It involves learning, but not in the more mechanistic and systematic mode of learning that is common in contemporary “system” quality thinking. Rather, learning is a way of experimenting; it is about probing and tinkering and learning is temporal and spatial. Rather than building protocols or procedures that can be implemented “elsewhere”, improvements are sought through situated connections and research and learning activities to improve a certain situation.

### Obeying, bending and ignoring organizational policies

Rebels and their clinical microsystems, we reveal, “go their own way”. Yet, they are also part of the hospital infrastructure shaped through, among other elements, insurance contracts, safety regulations supervised by external authorities (e.g. the healthcare inspectorate, audit bodies) and performance management systems. How does this work? In this section, we show how rebels obey, bend and ignore hospital quality policies and how they create spaces for doing so.

As indicated above, rebels relate quality of care to actual care encounters “clinical work” rather than protocols and measurement systems. Such rebellious work is about adjusting care to the needs of individual patients, rather than about following procedures. This included a critical attitude towards external performance management systems. In Hospital A, physicians and nurses alike appeared annoyed with the hospital’s focus on performance measurement. The ward manager argued that measuring needed to make sense, for instance, when scoring a vulnerable elderly patient who runs the risk of developing a delirium. Frequently scoring such a patient’s well-being enables health professionals to detect a shift in bodily functions and behaviour in time to act quickly if needed. Yet, he argued, other scores may not make sense from such a clinical quality perspective:

A striking example is fall prevention. [Following hospital’s policy] patients need to be informed about preventative measures through a letter if they have a higher fall risk. Well, we often have troubled patients in here who can’t even read and who may become suspicious if we give them the letter. We print out these letters as they are being counted as part of our performance dashboard, but then we throw them away. Tick the box.

(interview nurse manager, Hospital A)

At other times, however, ignoring or cosmetic compliance was not possible, and the rebels had to align with hospital policies, inducing another form of cosmetic compliance. Again, we exemplify with Hospital A:

During the accreditation period, we kept linen cloths and diapers out of the sink cupboard to meet the hygiene requirements. Normally we keep them in there, because we have a large population of patients with incontinence and wounds. These patients require a lot of care, and we want to facilitate this. Clean towels and linen cloths should always be near.

(senior nurse, Hospital A)

As these excerpts reveal, rebels tinker with rules; they deviate from them to live up to their own quality ambitions. If a hospital’s needs become more pressing, however, they obey the rules – temporarily at least. Furthermore, they seek to maintain positive relationships with colleagues and directors outside the clinical microsystem. For instance, the infectious disease ward of Hospital A was well known for its willingness to admit patients to their ward who were perceived as having caused problems elsewhere. This provided the rebels with some goodwill to deviate from rules as they were, in any case, seen differently among others due to their particular patient population anyway.

Tinkering with rules involves tinkering with visibility (Star, 1999; Timmermans *et al.*, 1998). Rebels have the tendency to “stay under the radar” to pursue their quality work. They seek to create space whilst safeguarding outside support and legitimacy. A clinical manager from Hospital B explained how she sometimes attempted to remain invisible:

I have the feeling that they [hospital board] want to control everything, every single word and comma […] I find that annoying; the feeling that they’re looking over my shoulder, that I need to account each and every time. Really, a shame that I have to waste my energy and time! […] So, if they don’t ask, I say nothing. If they ask a general question they’ll get a general answer. I conceal, because I think: everything they know more than strictly necessary may be used against me […].

(clinical manager Hospital B)

Rebels tend to conceal themselves from hospital directors seeking to control their practices. At the same time, paradoxically, rebels are highly visible and willing to account to an even larger public. All three clinical wards had recently received media attention, showing their abilities to provide high-quality care. They had been broadcast or written about in a popular news magazine. One of the managers had acted in a documentary. Hence, media coverage and broader publicity are a way of getting attention and “showing off” the exemplary work, thereby creating legitimacy for deviant action undertaken. Moreover, rebels were happy to account for what they were doing within the team and with external actors that they collaborated with in their “contexting work”. However, these ways of accounting did often not fit in with the hospital’s system approach of quality monitoring.

From our findings, it emerges that rebels like to do it their own way, but at the same time are very much aware of their organizational contexts, and take care of these as well. Rebels walk the tightrope in creating and safeguarding legitimacy and trust while tinkering with their visibility and quality aims. They make sure not to harm their organization and they nurture relationships, but at the same time they “cheat a bit” to stay close to their ideas and convictions. Moreover, such rebellion reveals community thinking, and a tendency to stay away from organizational control. Organizations, in turn, seem to tinker with their rebels: they are their positive examples and, at times, even their billboard as they are eager to present their achievements to the outside world. Yet, being rebels, they can also hard be to control. The quality manager in Hospital A admitted that this could be annoying. She stated that she would rather collaborate with clinical and nursing managers who tried to improve the management system than deviating from it. The hospital’s executive admitted that a rebel group can be a difficult one for the hospital management. Yet, he was also convinced of the group’s quality work and stated that he did sometimes better “not know” what this group was doing or not doing, as he then would have to explicitly stand against them. At the same time, he admitted the danger of groupthink due to lack of outside involvement which may make the organization blind to clinical errors.

## Discussion

The rebels portrayed in this paper like to do things somewhat differently. Steered by their intimate ways of clinical knowing and patient orientation, rebels put considerable effort into building and caring for their clinical microsystems. They define their own work area – materially, clinically and socially. In this research, we introduced the concept of “context work” and “contexting” to point out the professional and managerial practices of actively and continuously creating socio-material environments in which rebels could actively engage with actors within and outside their organizations to achieve aimed-for quality practices, such as safeguarding quality standards in case a manufacturer changes names for drugs, increasing the risk of errors in drug administration. Contexting thus takes shape both at the individual level of patients and at the level of organizing care. While in the literature, context is often depicted as something outside the control of actors, impinging on their practices, we have illustrated how rebel groups actively create contexts to do quality care and account for it. Furthermore, we have shown that contexting involves compelling normative work: rebels establish their own standards of optimal care and put a lot of effort in persuading others of those “goods”. The active work is both goal oriented and strategic. Rebels “walk the tightrope” to safeguard legitimacy and to avoid harming the organization they are ultimately a part of. Hence, rebels conduct boundary work (Gieryn, 1983) in the sense that they define boundaries and care for their own microsystem; defining, facilitating and protecting the work being done. Yet they also intentionally cross boundaries to create outside contexts that enable and facilitate quality work, allowing them to legitimately carry out their activities in the hospital environment.

In this paper, we have developed a situated approach to quality of care. Following this approach, quality is an emerging property rather than a well-defined and transferrable practice. Rebels’ quality practices are often temporal and spatial, rather than systematic and universal. Rather than a predefined and systematic way of acting and evaluation – which is common in the dominant system approach to patient safety in healthcare organizations as well as in the “positive deviants” literature – rebels undertake temporal and spatial activities of improving care. Such an approach emphasizes experimenting rather than knowing, and emerging rather than measuring and calculating (Clegg *et al.*, 2005; Mesman, 2012).

It is important to note that situatedness does not imply that rebels’ ways of doing cannot be transferred or copied; their approach to crafting microsystems and contexting can indeed be learned from as a quality approach to caring. Yet, their approach requires situated attention and work to create such quality environments. That is, while we have identified several mechanisms at play in the work that rebels do to create high-quality care, these are not “traits” that can easily be transferred, but rather form a situated culture of working that has to be developed over time.

Our situated approach to articulating quality rebels does explicitly involve re-evaluating the autonomous – or stubborn – clinician unwilling to account to “others”. On the contrary, we see rebels being highly willing to account for their work, but to do so in alternative ways, such as within the media and through the networks that they build, because their practices often fit uneasily with contemporary system quality thinking that exerts control over care provision (Hollnagel *et al.*, 2013; Pedersen, 2016). Moreover, accounting for clinical work in the rebel version is “generative” (Jerak-Zuiderent, 2013), in the sense that it is directly linked to the goals they are pursuing and tied into the networks that they create. Other forms of accounting, such as ticking boxes for performance information, are often ignored or only symbolically followed.

Our generative conception of quality rebels leaves us with some compelling organizational and management issues. Although the rebels portrayed in this paper may be quite exceptional cases (e.g. not all nursing wards make it into a newspaper article or TV documentary), they enable us to scrutinize existing quality regulations reflected in the contemporary patient safety debate. Contemporary quality thinking moves between “control” (such as in measuring through quality management systems) and “compassion”. They both rest on the belief that valuable ideas can be unearthed and moved somewhere else. Therefore, to some extent at least, both views still reflect a system or universal approach. Yet, the rebels in this paper work in a situated manner and can only do so through occasionally concealing some aspects of their work from hospital management systems. The risk is inducing a new danger of groupthink in which compelling beliefs may hide other needs or possible dangers. Although accountability is built into the probing and experimenting of rebel groups, these forms of accounting are not reflected in wider organizational quality machineries, and thus often stay below the radar of the organization.

This research has implications for policy and management. It shows that, in order to come to more compassionate forms of care quality management, organizations should allow for more heterogeneity accompanied with practices of supervision and ongoing dialogue(s) on what optimal care involves as this may differ between specific fields, locations or circumstances. This requires more situated and reflexive infrastructures of accounting. Clinical groups, comprising the various practitioners and managers involved, should rethink current system-based quality systems to unravel what elements contribute to optimal care, given the importance of some degree of accounting and control, and what impedes them developing alternative quality procedures. Such procedures could be focussed at creating more extended networks within the hospital organization to stimulate collective tinkering and reflexivity. These might include, for example, organizing mini audits based on forms of appreciative inquiry. Policy makers, hospital executives and quality staff members could facilitate such movement by actively encouraging clinical groups to develop situated and collective quality practices, and alternative measurements and ways of public accounting. Forms of process-based regulation, rather than standards-based regulation and oversight (e.g. Gilad, 2010; van de Bovenkamp *et al.*, 2014) might be helpful in creating such alternative and more generative ways of accounting.

This research has provided in-depth insight in how deviant and compassionate care are enacted in everyday clinical practice, and the caring craft work this involves. There are some limitations to this study, however. First, there was limited number of cases and the fact that the research was conducted in one country only. This research has underscored the situated enactment of rebellious care. Hence more insight is needed to further unravel this situatedness, and the contextual conditions that are required to provide care that is safe and of high quality. These conditions may moreover differ between countries as different institutional conditions may provide different opportunities and risks. Furthermore, a more extensive research approach in terms of time (observing rebels for longer periods of time) and choses time slots (observing more night and weekend shifts, for instance) may have revealed more tensions (e.g. between management and practitioners) and clinical risks that result from the way rebels act. Nevertheless, the sampling and data collection appear generally robust, because a relatively high number of actions, interactions observed and opinions conveyed in interviews, yielded the relatively limited and coherent set of conditions of the work of quality rebellion that were documented in this paper. Future research should focus more on rebels’ accounting practices and management-practitioner interactions.

## Figures and Tables

**Table I:** Details hospital case studies

	Interviews round I	Interviews during observation period	Interviews round III	Focusgroup
Hospital A	hospital executive, nurse director, chair medical staff, director quality department	Nurse manager, senior nurse, registered nurse (2), quality manager,	hospital executive nurse director, director quality department, chair calamity committee	
Hospital B	hospital executive, chair medical staff, chair and member nursing staff, director quality department, leading physician in the quality committee	nurse manager, medical manager, physicians (2), nurse practitioner, registered nurse (2), technician		hospital executive, nurse director, chair medial staff, chair nursing staff, director quality department, leading medical specialist in the quality committee
Hospital C	hospital executive, member medical staff focussing on quality and safety, chair nursing staff, director quality department, leading physician in the quality committee	nurse manager, medical manager, senior nurse (2), physicians assistants (2), registered nurse (3), specialized nurse		hospital executive, member medical staff focussing on quality and safety, chair nursing staff, director quality department, leading physician in the quality committee nurse manager, medical manager

## Notes

1. There are various terms to point out this group of actors who challenge existing quality systems by changing or adding quality to them: (social) deviants, positive deviants, vigilantes and (quality) radicals. These literatures primarily focus on the characteristics of the persons challenging the system. We use the term “rebel” and “clinical microsystem” to draw attention to the practices these actors enact; their own role as well as the socio-material environments they create.

2. E.g. one of the reasons why the executive of Hospital A pointed out the infectious diseases ward to us was because they had bought their own washing machine to be able to wash the clothes of the homeless so these people would at least have fresh clothes when being discharged from the hospital.

## References

[ref001] Asdal K. and Moser I. (2012), “Experiments in context and contexting”, Science Technology & Human Values, Vol. 37 No. 4, pp. 291-306.

[ref002] Berg M., Meijerink Y., Gras M., Goossensen A., Schellekens W., Haeck J., Kallewaard M. and Kingma H. (2005), “Feasibility first: developing public performance indicators on patient safety and clinical effectiveness for Dutch hospitals”, Health Policy, Vol. 75 No. 1, pp. 59-73. 16298229 10.1016/j.healthpol.2005.02.007

[ref003] Berwick D.M., Loehrer A. and Gunther-Murphy C. (2017), “Breaking the rules for better care”, Journal of American Medical Association, Vol. 317 No. 21, pp. 2161-2162. 10.1001/jama.2017.4703 28448652

[ref004] Bevan H. (2013), “A call to action: Helen Bevan’s blog”, available at: https://blogs-bmj-com.eur.idm.oclc.org/quality/2013/07/29/a-call-to-action-helen-bevans-blog/ (accessed 9 June 2019).

[ref005] Bonde M., Bossen C. and Danholt P. (2018), “Translating value-based healthcare: an experiment into healthcare governance and dialogical accountability”, Sociology of Health & Illness, Vol. 40 No. 7, pp. 1113-1126. 29675960 10.1111/1467-9566.12745

[ref006] Bozeman B. and Anderson D.M. (2016), “Public policy and the origins of bureaucratic red tape: implications of the Stanford yacht scandal”, Administration & Society, Vol. 48 No. 6, pp. 736-759.

[ref007] Bradley E.H., Curry L.A., Ramanadhan S., Rowe L., Nembhard I.M. and Krumholz H.M. (2009), “Reserach in action: using positive deviance to improve quality of health care”, Implementation Science, Vol. 4, p. 25. 19426507 10.1186/1748-5908-4-25 PMC2690576

[ref008] Clegg S.R., Kornberger M. and Rhodes C. (2005), “Learning/becoming/organizing”, Organization, Vol. 12 No. 2, pp. 147-167.

[ref009] Davies S.R. and Horst M. (2015), “Crafting the group: care in research management”, Social Studies of Science, Vol. 45 No. 3, pp. 371-393. 26477197 10.1177/0306312715585820

[ref010] Dixon-Woods M., Yeung K. and Bosk C.L. (2011), “Why is UK medicine no longer a self-regulating profession? The role of scandals involving ‘bad-apple’ doctors”, Social Science & Medicine, Vol. 73 No. 10, pp. 1452-1459. 21975027 10.1016/j.socscimed.2011.08.031

[ref011] Dixon-Woods M., Suokas A., Pitchforth E. and Tarrant C. (2009), “An ethnographic study of classifying and accounting for risk at the sharp end of medical wards”, Social Science & Medicine, Vol. 69 No. 3, pp. 362-369. 19535193 10.1016/j.socscimed.2009.05.025

[ref012] Gieryn T. (1983), “Boundary-work and the demarcation of science from non-science: strains and interest in professional ideologies of scientists”, American Sociological Review, Vol. 48 No. 6, pp. 781-795.

[ref013] Gilad S. (2010), “It runs high in the family: meta-regulation and its siblings”, Governance & Regulation, Vol. 4 No. 4, pp. 485-506.

[ref014] Helderman J.K., Schut F., Van Der Grinten T.E.D. and Van De Ven W.P.M.M. (2005), “Martket-oriented health care reforms and policy learning in the Netherlands”, Journal of Health Policy, Politics and Law, Vol. 30 Nos 1-2, pp. 189-210. 10.1215/03616878-30-1-2-189 15943393

[ref015] Hollnagel E., Braithwaite J. and Wears R.L. (Eds) (2013), Resilient Health Care, Ashgate, Farnham and Burlington.

[ref016] Huising R. (2014), “To hive or to hold? Producing professional authority through scut work”, Administrative Science Quarterly, Vol. 60 No. 2, pp. 263-299.

[ref017] Institute of Medicine (2000), To Err is Human, National Academies Press, Washington, DC.

[ref018] Jerak-Zuiderent S. (2013), “Generative accountability: comparing with care”, PhD, Erasmus University Rotterdam, Rotterdam.

[ref019] Kiers B. (2018), “Zorgprofessionals zetten mes in regelgekte zorg”, Zorgvisie, 27 March.

[ref020] Liberati E.G., Tarrant C., Willars J., Draycott T., Winter C., Chew S. and Dixon-Woods M. (2019), “How to be a very safe maternity unit: and ethnographic study”, Social Science & Medicine, Vol. 223, pp. 64-72. 30710763 10.1016/j.socscimed.2019.01.035 PMC6391593

[ref021] Mesman J. (2012), “Moving in with care: about patient safety as a spatial achievement”, Space and Culture, Vol. 15 No. 1, pp. 31-43.

[ref022] Mol A., Moser I. and Pols J. (2010), “Care: putting practice into theory”, in Mol A., Moser I. and Pols J. (Eds), Care in Practice: On Tinkering in Clinics, Homes and Farms, Transcript Verlag, Bielefield, pp. 7-25.

[ref023] Morrison E.W. (2006), “Doing the job well: an investigation of pro-social rule breaking”, Journal of Management, Vol. 32 No. 1, pp. 5-28.

[ref024] Nelson E.C., Batalden P.B., Huber T.P., Mohr J.J., Gofdfrey M.M., Headrick L.A. and Wasson J.H. (2002), “Microsystems in healthcare: part 1. Learning from high performing frontline clinical units”, International Journal of Quality Improvement, Vol. 28 No. 9, pp. 472-493. 10.1016/s1070-3241(02)28051-7 12216343

[ref025] Nicolini D., Mengis J., Meacheam D., Waring J. and Swan J. (2016), “Recovering the performative role of innovations in the global travel of healthcare practices”, in Swan J., Newell S. and Nicolini D. (Eds), Mobilizing Knowledge in Healthcare, Oxford University Press, Oxford, pp. 19-35.

[ref026] Pedersen Z.K. (2016), “Standardisation or resilience? The paradox of stability and change in patient safety”, Sociology of Health & Illness, Vol. 38 No. 7, pp. 1180-1193. 27397546 10.1111/1467-9566.12449

[ref027] Pope C. (2017), “A thing called Q”, Journal of Heallth Services Research & Policy, Vol. 22 No. 3, pp. 137-138. 10.1177/1355819617694919 28429982

[ref028] Power M., Scheytt T., Soin K. and Sahlin K. (2009), “Reputational risk as a logic of organizing in late modernity”, Organization Studies, Vol. 30 Nos 2-3, pp. 301-324.

[ref029] Singhal A. and Bjurström E. (2015), “Reframing the practice of social research: solving complex problems by valuing positive deviations”, International Journal of Communication and Social Research, Vol. 3 No. 1, pp. 1-13.

[ref030] Singleton V. and Mee S. (2017), “Critical compassion: affect, discretion and policy-care relations”, The Sociological Review Monographs, Vol. 65 No. S2, pp. 130-149.

[ref031] Star S.L. (1999), “The ethnography of infrastructure”, American Behavioral Scientist, Vol. 43 No. 3, pp. 377-391.

[ref032] Stoopendaal A. and Bal R. (2013), “Conferences, tablecloths and cupboards: how to understand the situatedness of quality improvements in long-term care”, Social Science & Medicine, Vol. 78, pp. 78-85. 23265806 10.1016/j.socscimed.2012.11.037

[ref033] Tarrant C., Leslie M., Bion J. and Dixon-Woods M. (2017), “A qualitative study of speaking out about patient safety concerns in intensive care units”, Social Science & Medicine, Vol. 193, pp. 8-15. 28987982 10.1016/j.socscimed.2017.09.036 PMC5669358

[ref034] Tavory I. and Timmermans S. (2014), Abductive Analysis: Theorizing Qualitative Research, The University of Chicago Press, Chicago, IL and London.

[ref035] Timmermans S., Bowker G.C. and Star S.L. (1998), “The architecture of difference: visibility, control and the comparability in building a nursing interventions classification”, in Berg M. and Mol A. (Eds), Differences in Medicine: Unraveling Practices, Techniques and Bodies, Duke University Press, Durham, NC, pp. 202-225.

[ref036] Tsoukas H. (2017), “Don’t simplify, complexify: from disjunctive to conjunctive theorizing in organization and management studies”, Journal of Management Studies, Vol. 54 No. 2, pp. 132-153.

[ref037] van de Bovenkamp H.M., De Mul M., Quartz J.G.U., Weggelaar-Jansen A.M.J.M.W. and Bal R. (2014), “Institutional layering in governing healthcare quality”, Public Administration, Vol. 92 No. 1, pp. 208-233.

[ref038] Wallenburg I., Quartz J. and Bal R. (2019), “Making hospitals governable: performativity and institutional work in ranking practices”, Administration & Society, Vol. 51 No. 4, pp. 637-663.

[ref039] Wallenburg I., Mol T., Harmsen M. and De Bruyne M. (2019), Onderzoek naar risicoselectie met de basisset kwaliteitsindicatoren ziekenhuizen: op weg naar verantwoorde keuzes, Amsterdam Public Health, Amsterdam.

[ref040] Wallenburg I., De Bont A., Heineman M.J., Scheele F. and Meurs P. (2013), “Learning to doctor: tinkering with visibility in residency training”, Sociology of Health & Illness, Vol. 35 No. 4, pp. 544-559. 23030786 10.1111/j.1467-9566.2012.01512.x

[ref041] Waring J. (2013), “What safety-II might learn from the socio-cultural critique of satey-I”, in Hollnagel E., Dekker S. and Braithwaite J. (Eds), Resilient Health Care, Ashgate, Surray and Burlington, NJ, pp. 39-48.

[ref042] Waring J.J. and Bishop S. (2010), “Water cooler learning: knowledge sharing at the clinical ‘backstage’ and its contribution to patient safety”, Journal of Health Organization and Management, Vol. 24 No. 4, pp. 325-342. 21033632 10.1108/14777261011064968

[ref043] Weggelaar-Jansen A.M.J., Broekharst D.S. and De Bruijne M. (2018), “Developing a hospital-wide quality and safety dashboard: a qualitative research study”, BMJ Quality & Safety, Vol. 27 No. 12, pp. 1000-1007. 10.1136/bmjqs-2018-007784 PMC6288703 29950323

[ref044] Zuiderent-Jerak T. and Berg M. (2010), “The sociology of quality and safety in health care”, in Bird C.E., Conrad P., Fremont A.M. and Timmermans S. (Eds), Handbook of Medical Sociology, Vanderbilt University Press, Nashville, TN, pp. 324-337.

